# Heart rate and sentiment experimental data with common timeline

**DOI:** 10.1016/j.dib.2017.10.037

**Published:** 2017-10-23

**Authors:** Jaromír Salamon, Roman Mouček

**Affiliations:** University of West Bohemia, Faculty of Applied Sciences, Department of Computer Science and Engineering, Univerzitní 8, 306 14 Pilsen, Czech Republic

**Keywords:** Sentiment analysis, Heart rate, Wearable technology, Soft and hard data relation, Common timeline

## Abstract

Sentiment extraction and analysis using spoken utterances or written corpora as well as collection and analysis of human heart rate data using sensors are commonly used techniques and methods. On the other hand, these have been not combined yet. The collected data can be used e.g. to investigate the mutual dependence of human physical and emotional activity.

The paper describes the procedure of parallel acquisition of heart rate sensor data and tweets expressing sentiment and difficulties related to this procedure. The obtained datasets are described in detail and further discussed to provide as much information as possible for subsequent analyses and conclusions. Analyses and conclusions are not included in this paper.

The presented experiment and provided datasets serve as the first basis for further studies where all four presented data sources can be used independently, combined in a reasonable way or used all together. For instance, when the data is used all together, performing studies comparing human sensor data, acquired noninvasively from the surface of the human body and considered as more objective, and human written data expressing the sentiment, which is at least partly cognitively interpreted and thus considered as more subjective, could be beneficial.

**Specifications Table**

**Table:** 

Subject area	Health Informatics, Health Science
More specific subject area	Using wearables and social media to collect hard and soft data for further usage in health science
Type of data	Text files (4 CSV files)
How data was acquired	Pilot experiment – 2×50 days, one participant, Twitter – sentiment data, Fitbit Charge HR, Basis Peak – heart rate data
Data format	Raw, Preprocessed
Experimental factors	Heart rate collected 24×7 together with min. 20 tweets recorded per day during 2× 50 days' experiments
Experimental features	The paper describes the procedure of parallel acquisition of heart rate sensory data and tweets expressing sentiment and difficulties related to this procedure.
Data source location	Zurich, Switzerland
Data accessibility	Data is provided with this article

**Value of the data**

• The data represents a mixture of soft (sentiment) and hard (heart rate) data collected in common timeline.
• The data can be used for independent analysis of heart rate time series (each experiment alone or both experiments together) or text sentiment extraction (also each experiment separately or concatenated into one corpus).
• Sentiment extraction can be done either by supervised methods (particular documents are annotated by positive – #p or negative – #n sentiment hash-tags) or unsupervised methods with further accuracy evaluation.
• The data are useful for further studies of relations between soft and hard data when joined together over the common timeline (as two independent experiments for the comparison of their outputs, or together to get a larger data collection).

## 1 Data

Human physical activity, its relation to emotional states and human sentiment expressed in a spoken or written form, all these phenomena are broadly scientifically investigated. With the widespread use of home and reliable monitoring devices and social networks, it is currently easy to collect data from them as well as collect texts expressing human feelings in the current or ongoing situation.

When we measure e.g. the steps during the day, heart rate, or respiration we get only the data, which provides us with one-dimensional information. Moreover, considering e.g. heart rate, we measure just the change of its values (heart rate increase/decrease) without understanding the reason (stress, excitement, deprivation, tiredness, etc.). This data can be enriched by additional data describing the context and collected simultaneously, such as human sentiment.

The overall purpose of the experiment is to map human physical activity and look for its influence on human feelings. This aim could be supported by hard (objective) data obtained from sensors capturing human physiological signals and soft (subjective) data written by people themselves.

More specifically, this article provides readers with the description of physiological data obtained from heart rate sensors and emotional data represented by tweets. Both of them were collected during a pilot experiment performed by one participant during two fifty-day periods. To our best knowledge, we do not know about such a source of data already been published.

### 1.1 Heart rate

The data structure for both datasets *experiment-1_fitbit.csv* and *experiment-2_basis.csv* is the following:

• experiment-1_fitbit.csv attributes:
  ○ Date_Time – CET date and time (format “YYYY-MM-DD HH:MI:SS”)
  ○ HR – heart rate [bpm] (numeric)
• experiment-2_basis.csv attributes:
  ○ Date_Time – CET date and time (format “YYYY-MM-DD HH:MI:SS”)
  ○ HR – heart rate [bpm] (numeric)
  ○ Steps – steps (numeric)
  ○ GSR – galvanic skin response (numeric)
  ○ Calories – burned calories (numeric)
  ○ Temp – skin temperature [°F] (numeric)

#### 1.1.1 Summary

While the text representing the participant's sentiment was recorded manually (the daily maximum was 23 tweets), the heart rate was measured using the selected monitoring devices. Their sampling frequency was strictly given. Both devices measured heart rate 24×7, but they had to be recharged after 4 to 5 days. The overview of the gathered data from both devices is available in Table 1.

**Table 1:** Summary of heart rate data.

	**Experiment #1 with Fitbit Charge HR**	**Experiment #2 with Basis Peak**
**Total number of samples**	411,799	69,941^a^
**Minimum number of samples per day**	6147	961
**Maximum number of samples per day**	9469	1440
**Average number of samples per day**	8074	1371
**Starting timestamp**	2016-05-10 00:00:05 CET	2016-08-15 00:00:00 CET
**Final timestamp**	2016-06-29 23:58:55 CET	2016-10-04 23:59:00 CET
**Device sampling frequency**	Every 5 s^b^	32 times per second^c^
**Device recording frequency**	Every 5–15 s	Every 1 min,
	i.e. 6–7 records per minute	i.e. 1 record per minute

a Only the samples where HR value is available (not equal to N/A), the total number of samples is 73,440.

b Information was obtained from a company representative response in the Fitbit community forum [14]

c Information was obtained from a company representative response in the Basis Peak forum [3] and [4] and from a prestige IT magazine [5].

#### 1.1.2 Gaps in data collection

The wearables used in the experiment had limited battery power and were not flawless. With knowledge of the frequency of data sampling, we can find and report gaps in the heart rate data collection.

The gap in the data collection from experiment #1 (using Fitbit Charge HR) is every time difference between subsequent records greater than 15 s (the longest recording time interval). In the data collection from experiment #2 (using Basis Peak) where the aggregated values per minute are available, we were looking for subsequent records with the time difference greater than 60 s.

We can divide the gaps found in the data collections into errors resulting from the process of data collection itself and into the gaps caused by recharging (Table 2). From the experiment perspective, the most interesting gaps are between 7:30 A.M. and 0:00 A.M. CET because these data can be analyzed along with the collected tweets.

**Table 2:** Summary of gaps in data collections.

	**Experiment #1 with Fitbit Charge HR**		**Experiment #2 with Basis Peak**	
	**7:30 A.M. – 0:00 A.M. CET**	**All-day**	**7:30 A.M. – 0:00 A.M. CET**	**All-day**
**Minor gaps (data errors) (> 15, <300 seconds)**	54	85	20	24
**Middle gaps (data errors) (>=300, <2700 s)**	7	10	0	0
**Major gaps (data errors) (>=2700, <10,000 s)**	6	6	0	0
**Charging (>=10,000 seconds)**	6	12	4	9
**Total**	73	113	24	33

**Fig. 1:**
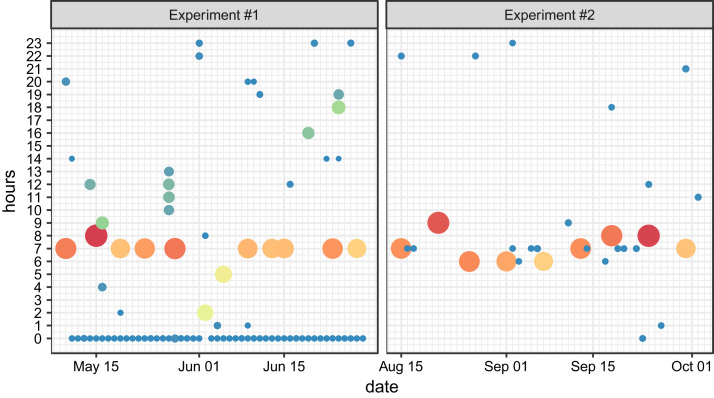
Gaps in the data per day and hour.

Fig. 1 shows the accumulated gaps in the data per day along with a specific hour the particular gap ended, i.e. the time difference between two HR timestamps (in seconds), for Fitbit Charge HR the threshold of 15 s is defined (the longest possible period between two measures):

#### 1.1.3 Heart rate precision measurement

Overall, the precision of the HR measurement is not crucial. However, for the purpose of the experiment, the tendency of the HR change at the moment of sentiment extraction is important. In the case of a systematic error occurring during the HR measurement, we would still get useful data collections. Only random errors or systematic errors appearing only in certain parts of the HR data could cause difficult data interpretation.

##### 1.1.3.1 Fitbit Charge HR

Considering the lawsuit to Fitbit regarding the product accuracy [9] we took into account information from many sources [11], [12], [13]. One of the sources [10] providing a lot of information about Fitbit is cited but seems to be anecdote research.

However, the results from [12] show that the mean absolute percentage error was 6.2% when the comparison between Fitbit Charge and ECG was done. Other results from [13] present that device heart rate estimates were within 1–9% of reference estimates.

##### 1.1.3.2 Basis Peak

The precision of the HR measurement for the entire testing interval was determined as an average difference of 3.6% between the values measured by the Basis Peak and the ECG. The HR values were produced over 99.5% of the testing period [2]. This precision is also confirmed by the results of [12], which presents the mean absolute percentage error of 3.6% during its comparison to ECG.

### 1.2 Twitter corpus

Both twitter corpora have the same description of columns in the provided datasets:

• *experiment-1_twitter.csv*, *experiment-2_twitter.csv*:
  ○ Day_Pos - twitter order during the day (numeric)
  ○ Date_Time - CET date and time (format “YYYY-MM-DD HH:MI:SS”)
  ○ Date_Time_Exp - CET date and time when the tweet was expected (format “YYYY-MM-DD HH:MI:SS”), see Section 2.1.3 *Sentiment expressed in tweets*
  ○ Sent_Num - evaluated sentiment (numeric), represented by 1 = positive, -1 = negative extracted from the text hash tags (#p = positive, #n = negative)
  ○ Text – the original text of tweet including @ and # (varchar max. 140 characters)

#### 1.2.1 Summary

The data representing the sentiment that was collected during the 50 (the data includes tweets from 51 days) days’ experiment is summarized in Table 3. The positive and negative sentiment is not extracted by machine learning methods; it is evaluated by the participant and recorded using the positive and negative hashtags.

**Table 3:** Summary of the corpus data per tweet.

	**Experiment #1 with Fitbit Charge HR**	**Experiment #2 with Basis Peak**
**Total number of tweets**	1029	1017
**Number of positive tweets**	780	718
**Number of negative tweets**	249	299
**Average number of tweets per day**	20.56	20.32
**Positive to negative ratio**	3.13:1	2.40:1
**Number of days with 20+ tweets**	35/50 (70%)	35/50 (70%)
**Date and time of the first tweet**	2016-05-10 07:36:17 CET	2016-08-15 10:01:24 CET
**Date and time of the last tweet**	2016-06-29 01:10:15 CET	2016-10-04 00:03:35 CET

#### 1.2.2 Deviations from expected tweet creation time

The process of writing tweets experienced from the first version of the experiment realized and described in [1].

The first crucial problem was a distribution of tweets during the day. For this purpose, the daily windows were used. More specifically, a tweet should have been written in the following times: 7:30, 8:15, 9:00, 9:45 … 22:30, 23:15, and 0:00. The exact time points were not strictly enforced, but the participant had a tendency to keep them.

**Fig. 2:**
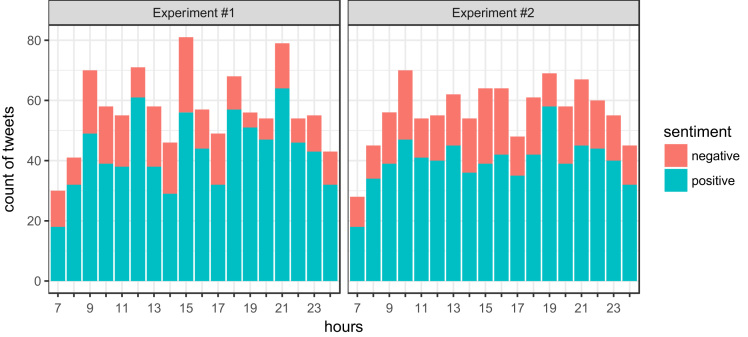
Distribution of tweets per hour for both experiments.

Fig. 2 shows how precise the participant was in tweeting during the experiment. As we can see the tweets are distributed evenly from 9:00 to 23:00 in both experiments. The values close to midnight are lowered by the fact that tweets were usually recorded after the midnight of the next day. Lower values are expected from 7:00 to 9:00 since tweets were not recorded during this time interval at the weekends.

**Fig. 3:**
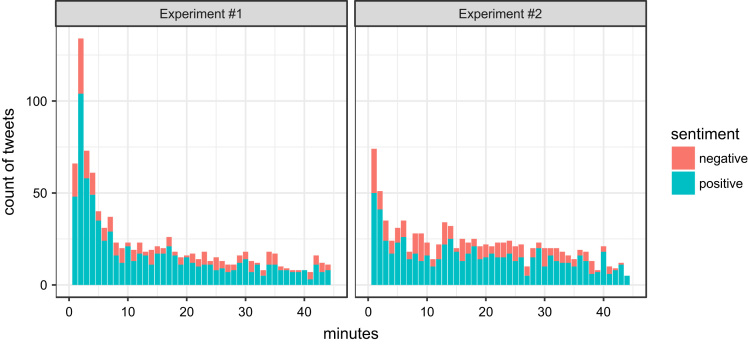
Deviations between real tweeting time and expected tweeting time.

The deviations from the expected times (the alarm announced the tweeting time every 45 min 21–23 times per day during the experiment duration) of particular tweets are illustrated in Fig. 3 for both experiments. As we can see, most tweets were written within the first 5 min after the expected time.

### 1.3 Time zone matrix

Since the experimental data is collected from three different data source systems working in different time zones, it is necessary to clarify which time zone was used during each data processing phase (see Table 4).

**Table 4:** The time zone matrix for a particular data source system and specific data processing phases.

	**Twitter API**	**Fitbit Charge HR API**	**Basis Peak API**
**Source time zone**	UTC	CET	UTC
**Exported data time zone**	CET	CET	UTC
**Provided data time zone**	CET	CET	CET
**Data analysis time zone**	CET	CET	CET

All three sources (Table 4) were carefully investigated and based on the knowledge of the particular activity the time zone was determined.

Table 4 clearly explains several phases when data was extracted and preprocessed and provides information what time zone is expected to be used during data descriptive analysis to avoid misunderstanding and pitfalls.

## 2 Experimental design, materials and methods

### 2.1 Experimental design

#### 2.1.1 Common part

Since two different wearables for the HR measurement were used, the experiment was performed in two periods, each with duration of 50 days:

• the first time period was from May 10th to June 28th, 2016,
• the second-time period was from August 15th, 2016 to October 3rd, 2016.

#### 2.1.2 Heart rate measurement

The wearables broadly available on the market were chosen. The main requirements were defined as follows:

• possibility to measure heart rate 24×7,
• data export in required granularity based on the sampling frequency,
• sampling frequency of output data is at least one sample per minute, but it is preferred to be higher.

**Fig. 4:**
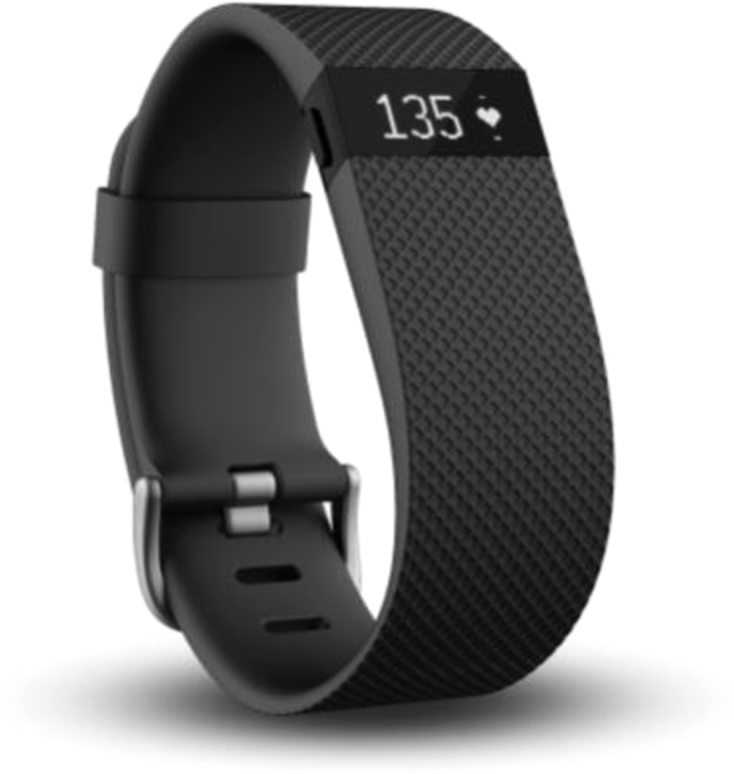
Fitbit Charge HR wearable from Fitbit company. Picture sourced from Fitbit press release kit: https://investor.fitbit.com/press/press-kit/charge-hr/default.aspx.

**Fig. 5:**
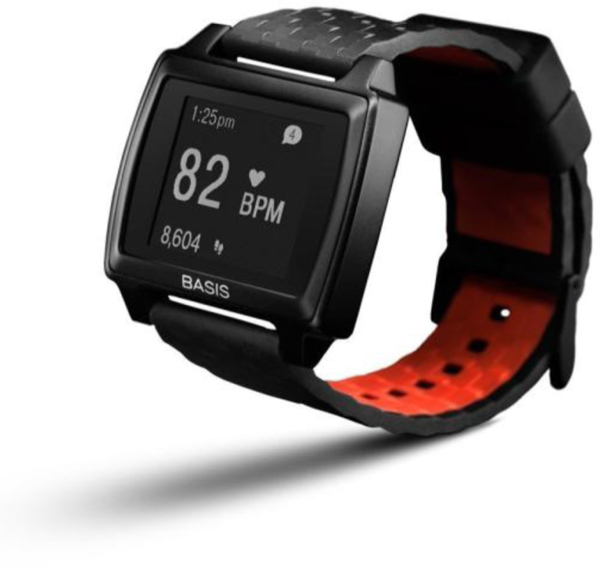
Basis Peak wearable from Basis company. Picture sourced from YouTube library (Peak is produced by Basis Company anymore): https://i.ytimg.com/vi/zhBYMR8t4_Y/maxresdefault.jpg.

During the market research and parameters evaluation, 30 devices from 16 companies were taken into account and two of them complied with the requirements (Figs. 4 and 5):

• FitBit Charge HR^1^ and
• Basis Peak.

#### 2.1.3 Sentiment expressed in tweets

The sentiment of the participant was expressed by writing short texts – tweets. The social network Twitter was used as a recording medium. It provides the ability to record the text together with its timestamp using a mobile phone with social media account and several appropriate applications. On the other hand, Twitter uses 140-character limit for text entry. It was considered as an advantage in our case because the participant had to express his/her sentiment briefly.

The text message was extended by two hash-tags (i.e. the words starting with #). The first one was used to identify the tweets related to the experiment:

• #xfb – eXperiment Fitbit (Charge HR)
• #xpb – eXperiment Basis Peak

These serve to simplify searching, filtering and exporting documents from Twitter.

The second pair of hashtags identifies participant's positive or negative mood during the tweet recording:

• #p – positive mood
• #n – negative mood

This can be used to facilitate the sentiment extraction.

The main daily window for tweeting was set to:

• 7:30 A.M. CET to 0:00 A.M. CET for working days,
• 9:00 A.M. CET to 0:00 A.M. CET for weekdays.

One tweet per 45 min was expected in these two defined windows. It means 23 tweets were expected during a working day and 21 tweets were recorded during a weekday. However, at least 20 tweets per day were required.

#### 2.1.4 Participant

The measured participant was a 35-years healthy man with treated high blood pressure having a heart rate of 94 bpm during regular physical activity, average heart rate of 72 bpm during physical inactivity (rest HR), and average heart rate of 100 bpm during higher physical activity (running, fast walking).

### 2.2 Working with common timeline

This section introduces the work with the common timeline for the obtained data. This idea was already described in our previous publication [1].

#### 2.2.1 API time precision

All servers providing particular web services (Twitter, Fitbit, Basis) are connected to the Internet and thus we assume their current time is synchronized through the network time protocol (NTP) or simple network time protocol (SNTP) [6]. NTP can usually set the time within tens of milliseconds over the public Internet and can achieve better than one millisecond accuracy in local area networks under ideal conditions. Asymmetric routes and network congestion can cause time precision errors of 100 ms or more.

#### 2.2.2 Merging datasets

The datasets for both experiments were acquired from four different data sources, so it would be convenient to have them in one dataset for each experiment. It is suitable to merge the data to have heart rate data and corresponding sentiment data together.

##### 2.2.2.1 Fitbit Charge HR dataset

Fitbit provides data with irregular granularity between 5 and 15 s (as it is described above). It means that for each tweet date and time attribute the entry in the heart rate data has to be found. We looked for the lowest absolute time differences. The final implementation for this experiment is based on the Python [7] and R [8] examples.

##### 2.2.2.2 Basis Peak dataset

In case of Basis, we did not deal with irregular granularity. The heart rate was provided in the aggregated form as the average per minute. So, datasets can be merged removing the seconds’ part from the tweets timeline.

##### 2.2.2.3 Gaps in merged data

When we merged twitter and HR datasets, there were always gaps in the merged data caused by the gaps in the HR dataset (see Section 1.1.1 *Gaps in data collection*). The method finding out the number of the twitter records that were lost (due to the gaps in the HR data) differs for both experiments.

For experiment #1, tweets met a gap whenever the time differences overreached some threshold. Since the HR data for Fitbit Charge HR was produced every 5–15 s, we defined the time differences greater than 15 s as the threshold.

For experiment #2 the gaps in the HR data were marked as NA values.

In experiment #1 we lost four tweets which did not match any HR values. In experiment #2 we did not lose any tweet.

### 2.3 Different granularity

When merging datasets, we get results with different granularity. This has to be considered during further data processing or analysis. A simple way of data processing is to get a single value of heart rate corresponding to its sentiment data has or resample sentiment data to achieve the same granularity as the heart rate data has.

The operations done on the data can be:

• Repeat the sentiment value for every HR value
• Calculate average, max or use other operation overall HR values belonging to the specific sentiment value within the defined interval

#### 2.3.1 Interval definition

**Fig. 6:**
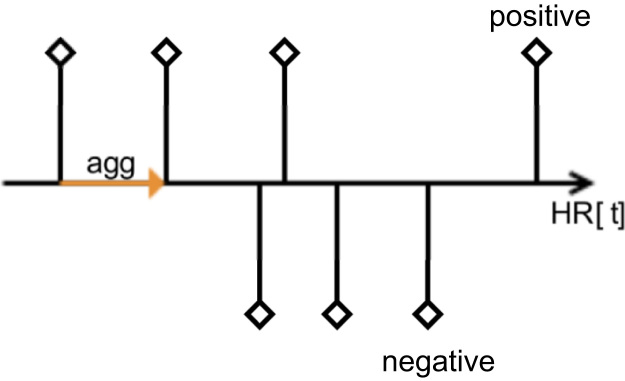
HR data aggregation between two sentiment values.

**Fig. 7:**
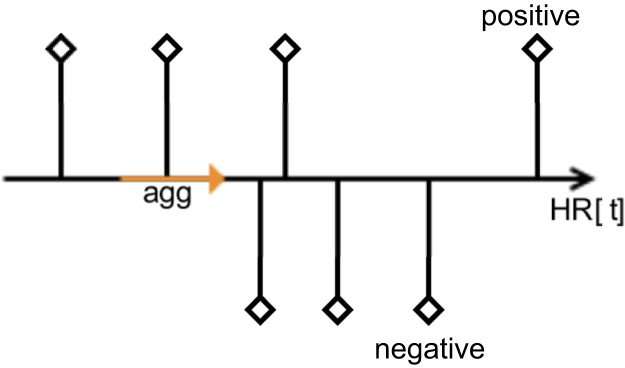
HR data aggregation over sentiment values.

To achieve the same granularity of the heart rate data and sentiment data we need to define an interval for heart rate data aggregation. We can either aggregate all values of the HR data between two specific sentiment values (see Fig. 6) or we can take an interval around a specific sentiment value to aggregate all values of the HR data falling into this interval (see Fig. 7).

## Footnotes

1 There are alternatives from Fitbit, e.g. Surge, Blaze or Charge 2, but when alternatives exist, we took the cheapest one.
